# Supplementary material for: Evaluation of innovation primacy in cross-regional central cities: Evidence from the Huaihai Economic Zone in China (2010–2020)
Source: PLoS One. 2024 Mar 8;19(3):e0299951. doi: 10.1371/journal.pone.0299951 (PMC10923477; doi:10.1371/journal.pone.0299951)
Supplement: Attachment — Submitted filename: Review PONE-D-23-33941.docx [file pone.0299951.s001.docx]

Review Report for Manuscript ID: PONE-D-23-33941. “Evaluation on the Innovation Primacy in Cross-regional Center Cities: Evidence from Huaihai Economic Zone in China (2010-2020)” I applaud the authors for their efforts in crafting this article. However, there are a number of issues with this paper that need to be revised. These concerns are delineated as follows:

1 In the first line of page 2, the author omitted a little information. Like, “In recent years, scholars such as [9,10]have gradually focused on the primacy of innovation.”. There are many other similar mistakes in this article.

2 Could you provide a detailed comparative analysis of your study's empirical results in relation to findings from existing literature. Highlight the distinctive aspects or divergences in your results compared to other studies, offering an in-depth empirical discussion to contextualize and interpret these differences.

3 Please give a mechanistic analysis of this conclusion. “Xuzhou can promote the improvement of the innovation capability of surrounding cities through the innovation leadership effect.”

**My Recommendation**: Minor Revision
