# Supplementary material for: Evaluation of innovation primacy in cross-regional central cities: Evidence from the Huaihai Economic Zone in China (2010–2020)
Source: PLoS One. 2024 Mar 8;19(3):e0299951. doi: 10.1371/journal.pone.0299951 (PMC10923477; doi:10.1371/journal.pone.0299951)
Supplement: Attachment — Submitted filename: Review PONE-D-23-33941.docx [file pone.0299951.s002.docx]

**Responses to the Reviewers**

**Reviewer #1:** Review Report for Manuscript ID: PONE-D-23-33941. “Evaluation on the Innovation Primacy in Cross-regional Center Cities: Evidence from Huaihai Economic Zone in China (2010-2020)” I applaud the authors for their efforts in crafting this article. However, there are a number of issues with this paper that need to be revised. These concerns are delineated as follows:

1. In the first line of page 2, the author omitted a little information. For example, “In recent years, scholars such as [9,10] have gradually focused on the primacy of innovation.”. There are many other similar mistakes in this article.

2. Could you provide a detailed comparative analysis of your study's empirical results in relation to findings from literature? Highlight the distinctive aspects or divergences in your results compared to other studies, offering an in-depth empirical discussion to contextualize and interpret these differences.

3. Please give a mechanistic analysis of this conclusion. Xuzhou promoted the improvement of the innovation capability of surrounding cities through the innovation leadership effect.

My Recommendation: Minor Revision

**Re:**

1. Thank you for your valuable comments. In the latest manuscript, I have perfected the details of the errors regarding the citations of the references in the text.

2. There are many studies on the leading effect of innovation primacy, but scholars have paid more attention to the development of leading city clusters or economic zones. The main purpose of the empirical section is to examine whether the innovation primacy of Xuzhou is effective and to provide a reference for future policy focus.

The research area of this paper is the Huaihai Economic Zone, which is described in the introduction section. The Huaihai Economic Zone is a bridge connecting the "Belt and Road Initiative" and the Yangtze River Economic Belt; it is located in the junction area of four provinces, namely, Su, Lu, Henan and Anhui. This zone has convenient traffic, a dense population and rich resources and is an important national base of food, energy and minerals, and manufacturing industry. This zone occupies an important national base in the overall situation of China's economic and social development, but to a certain extent, there is a large gap between it and world-class city clusters such as the Yangtze River Delta city cluster and the Guangdong-Hong Kong-Macao Greater Bay Area. Therefore, in general, based on the construction of plates and "circles", the construction of the Huaihai Economic Zone should break administrative divisions and market barriers. The "four new economies" of new technologies, new industries, new modes and new business forms were activated, the transformation of traditional industrialization to new industrialization was actively promoted, and high-tech industries were vigorously developed to make the Huaihai Economic Zone a nationally important science and innovation town. At present, the economic development of various industries cannot be separated from the positive externalities brought about by public products such as talent, education and medical care, in which innovation is the driving force. According to the theory of regional agglomeration, an a priori condition for regional agglomeration is to exploit the driving effect of the innovative development of the central city on the surrounding cities. Balanced development is meaningful to human civilization, and the current research on the Huaihai Economic Zone mainly comes from horizontal projects of industry-academia-research institutes. However, there is a lack of systematic research on economically underdeveloped regions. This paper is the first to focus on the region's performance in terms of innovation-leading effects; therefore, although we try to provide a reference for all developing regions with similar characteristics, the results may not necessarily be sufficient to generalize these differences.

3. Thank you for your comments, which are very interesting. We have performed a corresponding mechanism analysis for Xuzhou's innovation-leading effect, which is discussed in the submitted revision.

To further answer the question of what mechanism Xuzhou's innovation leadership effect can improve the innovation capacity of neighboring cities, according to value chain theory, we tested the effect of innovation capacity in three stages: (v) knowledge innovation primacy, (vi) R&D innovation primacy, and (vii) industrial innovation primacy. Additionally, we analyzed the role of the innovation leading effect channels from the (viii) industrialization level.

The leading role of Xuzhou in innovation is reflected in enhancing the leading position of surrounding cities in knowledge innovation, R&D innovation and industrial innovation and thus improving the innovation level of surrounding cities. In addition, enhancing the industrialization level of surrounding cities effectively promotes their innovation capacity.

**Table 5** Further results for innovation-led core cities in the Huaihai Economic Zone

| Variables | （5） | （6） | （7） | （8） |
| --- | --- | --- | --- | --- |
| Innovled | 0.0374^***^ | 0.0202^***^ | 0.0263^***^ | 0.0452^***^ |
|  | (0.0071) | (0.0040) | (0.0065) | (0.0124) |
| rpgdp | 13.1981^***^ | -1.7950 | -3.8009 | -2.8812 |
|  | (4.2010) | (2.3764) | (3.8308) | (7.3532) |
| io | -13.6493^*^ | 4.8973 | -13.0726^**^ | -22.1539^*^ |
|  | (6.8516) | (3.8758) | (6.2479) | (11.9928) |
| fin | 1.5344 | -9.1140^***^ | -10.0375^*^ | 47.7604^***^ |
|  | (5.8296) | (3.2977) | (5.3160) | (10.2040) |
| tech | 1.5344 | -9.1140^***^ | -10.0375^*^ | 47.7604^***^ |
|  | (5.8296) | (3.2977) | (5.3160) | (10.2040) |
| Constant | 4.8847^*^ | 2.5557^***^ | 7.3159 | 5.4172^*^ |
|  | (2.7784) | (0.6830) | (7.4986) | (2.7132) |
| i.city | control | control | control | control |
| i.year | control | control | control | control |
| N | 77 | 77 | 77 | 77 |
| R^2^ | 0.8904 | 0.9489 | 0.9281 | 0.9468 |

The leading role of Xuzhou in innovation is reflected in enhancing the leading position of surrounding cities in knowledge innovation, R&D innovation and industrial innovation and thus improving the innovation level of surrounding cities. In addition, enhancing the industrialization level of surrounding cities effectively promotes their innovation capacity. Specifically, (i) knowledge innovation primacy refers to the degree to which a city leads in terms of knowledge output, transformation of scientific and technological achievements and talent attraction. Xuzhou city can help its neighboring cities improve their knowledge innovation capacity by establishing cooperative alliances with research institutions, universities and science and technology enterprises to carry out technological exchanges and talent cultivation. (ii) R&D innovation primacy refers to the degree to which a city is led in terms of scientific research investment, technological innovation and product development. Xuzhou city can cooperate with enterprises in neighboring cities to carry out joint R&D projects and share technological resources to enhance the R&D innovation capacity of neighboring cities. (iii) Industrial innovation primacy refers to the degree to which a city leads in industrial structure adjustment, emerging industry cultivation and industrial upgrading. Xuzhou city can guide the enterprises in neighboring cities to carry out industrial upgrading and transformation through platforms such as industrial parks and science and technology parks to promote the improvement of the industrial innovation capacity of neighboring cities. (iv) The level of industrialization refers to the degree of industrialization and the level of industrial development of the city. Regions with high industrialization levels may be more likely to have advanced production technologies and management experience, thus promoting the enhancement of innovation capacity. In some cases, overindustrialization may lead to the overconcentration of resources and the inhibition of innovation capacity, resulting in a negative relationship. In the context of the Huaihai Economic Zone, the current level of industrialization has not yet reached a value inflection point. Xuzhou city can help its neighboring cities upgrade their industrialization level by introducing advanced production technologies and management experience, which will promote industrial upgrading and innovative development in the whole region and thus play a leading role in innovation.

As a result, the mediating effect of the industrialization level is reflected. In the future, Xuzhou city should increase in size, accelerate the improvement of strength, hub and radiation advantages, and make the advantages of being the first city more obvious and more influential.

**Reviewer #2:** 1. The description of the background of the article should be strengthened to emphasize why the topic should be studied.

2. To ensure the robustness of the empirical results, this paper should consider endogeneity and other issues.

3. This paper should analyze the limitations of this paper and put forward prospects at the end of the paper.

**Re:**

1. Thank you for your helpful comments. We have enhanced the description of the context and purpose of the study, specifically in the text.

In 2017, Xuzhou was planned and deployed as a separate important plate in the province's "1+3" functional area strategy, and opinions were issued to support the construction of the central city of Xuzhou in the Huaihai Economic Zone. The ability of the Huaihai Economic Zone to promote coordinated development at the national level is highly strategic for overcoming administrative barriers, promoting the "rise of depression", and supporting coordinated national innovation development. The Huaihai region is densely populated with small and medium-sized towns, and the linkages and cooperation between towns are extensive and close; Xuzhou, as the center of the regional town system, is becoming increasingly prominent. In 2022, the total number of Xuzhou's seven major economic indicators will be at the forefront of the 19 cities in the Huaihai Economic Zone. The city's GDP will reach 845.784 billion yuan, ranking first in the region and accounting for 11.7% of the regional share; retail sales of consumer goods will reach 410.273 billion yuan, ranking first in the region and accounting for 12.7% of the regional share; the balance of RMB deposits in financial institutions will reach 105.5362 billion yuan, ranking first in the region and accounting for 10.3% of the regional share; and the pattern of regional development centered on Xuzhou will be accelerated.

Therefore, it is highly important to strongly support the construction of Xuzhou as the central city of the Huaihai Economic Zone, to enhance the comprehensive function and innovation primacy of Xuzhou's central city, and to actively tilt toward Xuzhou in terms of coordinating the study of the major infrastructure and major productivity layout and the major carriers and platforms to further enhance the function of radiation and drive the regional central city of Xuzhou and to practically lead the collaborative and innovative development of the Huaihai city cluster.

2. Thank you for your kind suggestions, which are valid. The endogeneity issue is an issue that needs to be considered in each piece of empirical evidence; it can even be said to be the primary issue, which is important for the robustness of the results, and of course, we have done other elements of the analysis of the robustness of the empirical results.

A two-way fixed effects model is a statistical model that controls for fixed effects in panel data and can be used to control for externalities and exclude endogeneity. This model is able to control for the fixed effects of both individual and temporal characteristics, thereby reducing the effects of endogeneity. In empirical studies, two-way fixed effects models are commonly used to address endogeneity and omitted variables in panel data. Moreover, according to related studies, innovation primacy and innovation leadership effects do not suffer from endogeneity.

3. We have reanalyzed the limitations of this paper in our research. Thank you for your comments, which are important for the structural integrity of the article and for the readers to read and deepen this study.

Although some improvements have been made, due to data collection problems, there are still some blind spots that have not been popularized, such as the introduction of talent. Factors such as the input of innovative resources have not been well reflected in the text. Second, due to the problem of its source, the data in this paper are not updated to include the latest 2022 data, and some of the latest policies of Xuzhou city are not included in the text. Third, this paper does not study all the cities in the overall region of the Huaihai economy; rather, only the cities in the core area of the Huaihai economic region are the research object, and the choice is somewhat narrow. The Huaihai economic zone is one of the earliest regional economic cooperation organizations in China. It consists of twenty prefecture-level cities in Jiangsu, Shandong, Henan and Anhui Provinces. This study selected eight cities in the core area of the Huaihai Economic Zone as representatives for research, both because of the availability of data and workload factors and because of the special status of the core area of the Huaihai Economic Zone. The geographical location of cities in the core area is important, but the region is located in the economic depression zone of the four provinces. Accelerating the development and revitalization of a region is very important for promoting the coordinated development of the regional economy. In view of the above limitations, follow-up studies will further expand the breadth of data collection and update the existing data around these issues.

**Reviewer #3:** The author should pay more attention to the expression. It will be better with polish. The References list is not consistent in format. Some missing information needs to be curated when retrieved from the database. Most of the work is declarative, lacking author's comments. Open questions should be proposed for future research. Send to a more oriented journal would be more suitable.
Thank you for this comment.

**Re:**

Thank you for your valuable suggestions. We will focus on improving the presentation and further refining the content. We have retouched the manuscript with proof of retouching.

Moreover, we ensured that the format of the "citation" list was consistent, and we removed the missing information from the database search.

In addition, we will add more personal comments and ask open questions for future research. Your suggestions are very pertinent, and we have taken them on board. Thank you again for your guidance.

Although some improvements have been made, due to data collection problems, there are still some blind spots that have not been popularized, such as the introduction of talent. Factors such as the input of innovative resources have not been well reflected in the text. Second, due to the problem of its source, the data in this paper are not updated to include the latest 2022 data, and some of the latest policies of Xuzhou city are not included in the text. Third, this paper does not study all the cities in the overall region of the Huaihai economy; rather, only the cities in the core area of the Huaihai economic region are the research object, and the choice is somewhat narrow. The Huaihai economic zone is one of the earliest regional economic cooperation organizations in China. It consists of twenty prefecture-level cities in Jiangsu, Shandong, Henan and Anhui Provinces. This study selected eight cities in the core area of the Huaihai Economic Zone as representatives for research, both because of the availability of data and workload factors and because of the special status of the core area of the Huaihai Economic Zone. The geographical location of cities in the core area is important, but the region is located in the economic depression zone of the four provinces. Accelerating the development and revitalization of a region is very important for promoting the coordinated development of the regional economy. In view of the above limitations, follow-up studies will further expand the breadth of data collection and update the existing data around these issues.
